# Editorial for Special Issue “Advance in Diagnostic and Management of Ischemic Heart Disease”

**DOI:** 10.3390/diagnostics13061185

**Published:** 2023-03-20

**Authors:** Maria Dorobanțu

**Affiliations:** Faculty of Medicine, University of Medicine and Pharmacy “Carol Davila”, 050474 Bucharest, Romania; maria.dorobantu@gmail.com

Ischemic heart disease is one of the leading causes of morbidity and mortality worldwide. Continuous advances in diagnostic, prognostic and therapeutic methods have led to an improvement in patient outcomesover the past few decades.

In recent years, developments in molecular assessments, laboratory assays and imaging techniques have enabled a comprehensive characterisation of ischemic heart disease. Circulating biomarkers involved in the pathogenesis of CAD (coronary artery disease) have the potential to detect the disease in its early stages: inflammatory markers, biochemical markers (proteins), epigenetic markers (miRNA, ncRNA), and transcriptional markers (gene expression) [1]. Cardiovascular imaging techniques have enhanced our knowledge regarding physiological aspects and myocardial implications of CAD [2]. Echocardiography is an essential imaging modality in patients with ischemic heart disease (IHD) [3]. The assessment of LV mechanics through 3D speckle tracking echocardiography, LV-strain-derived myocardial research, and LA strain and stress echocardiography are a few of the most recent and notable ultrasound technologies [3]. Other imaging techniques have improved the early detection of CAD: intracoronary ultrasound (IVUS), coronary angiography and computed tomography coronary angiography provide a direct evaluation and quantification of coronary artery plaques, while cardiac magnetic resonance and nuclear medicine techniques (SPECT and PET) provide indirect information on CHD, estimating the myocardial perfusion and metabolism abnormalities secondary to ischemic heart disease [1].

This Special Issue explores some of the latest research on the diagnosis of coronary artery disease.

Calmac et al. [4] discuss the importance of visual estimation of coronary artery stenoses and the use of a functional evaluation for appropriate guidance on coronary revascularisation. A visual evaluation of coronary stenoses angiographic appearance (also known as eyeballing) by an interventional cardiologist represents the first evaluation step during invasive coronary angiography (ICA). Eighty-six patients in need of invasive functional evaluation were assessed in this study. An FFR measurement utilizing the intracoronary administration of adenosine was used as golden standard for defining the functional significance of the stenoses. Coronary revascularisation was determined based on clinical and invasive evaluations (including the FFR measurements), using clinical practice guidelines recommendations. The average accuracy of the visual prediction of functional significance of intermediate degree stenosis was 55%, advocating against the sole use of VE to support the decision of revascularisation and highlighting the importance of functional evaluation [4].

An important section of this Special Issue is dedicated to the implications of myocardial bridge in patients with CAD [5]. The myocardial bridge (MB) represents the muscle fibres that abnormally overlie the intramyocardial passage of an epicardial coronary artery, which consequently becomes tunnelled in its path beneath them. Although few isolated MBs have a clinical expression, myocardial ischemia induced by isolated MBs may manifest in significant clinical forms, such as silent ischemia, stable angina, acute coronary syndromes, arrhythmias, and even sudden cardiac death. Darabont et al. [5] studied 1920 consecutive patients who underwent coronary angiographyand identified 76 patients with myocardial bridging. Interestingly, the presence of MB on LAD is not an additional risk factor for coronary atherosclerosis. On the contrary, it proved to be a protective factor against atherosclerosis for the entire artery and for each of its segments, especially in its middle and proximal segments; however, it does not influence 10 year survival in patients with CAD [5].

Finally, the systematic review by Kampmann et al. [6] addresses an important diagnostic issue: acute myocardial infarction in patients with impaired renal function. Identifying acute myocardial infarction in patients with renal disease is difficult due to atypical presentation and chronically elevated troponin. This review aimed to identify a specific troponin T/troponin I cut-off value for the diagnosis of acute myocardial infarction. A total of 2590 publications were screened, 15 studies were included inthe qualitative synthesis, and only 6 were eligible for a meta-analysis. The cut-off values for troponin in patients with renal impairment with myocardial infarction were 42 ng/L for troponin I and 48 ng/L for troponin T. For patients on dialysis the troponin T cut-off is even higher at 240 ng/L. These optimised values will aid clinical assessment and thorough anamneses in diagnosing AMI in patients with impaired renal function [6].

All papers presented in this Special Issue emphasise the importance of diagnostic techniques in CAD allowing for patient-tailored treatment, and thus reducing ischemic-heart-disease-related complications and improving patient outcomes. Advances in diagnostic techniques and the management of these patients may substantially reduce the burden of cardiovascular diseases in the future.
